# Supplementary material for: The interactome of intact mitochondria by cross-linking mass spectrometry provides evidence for coexisting respiratory supercomplexes
Source: Mol Cell Proteomics. 2017 Dec 8;17(2):216–32. doi: 10.1074/mcp.RA117.000470 (PMC5795388; doi:10.1074/mcp.RA117.000470)
Supplement: Supplemental Data [file supp_RA117.000470_133922_0_supp_23978_fzffwf.zip › spectra_annotation/mito_spectra_annotation/251-1-3-1-2-1.pdf]

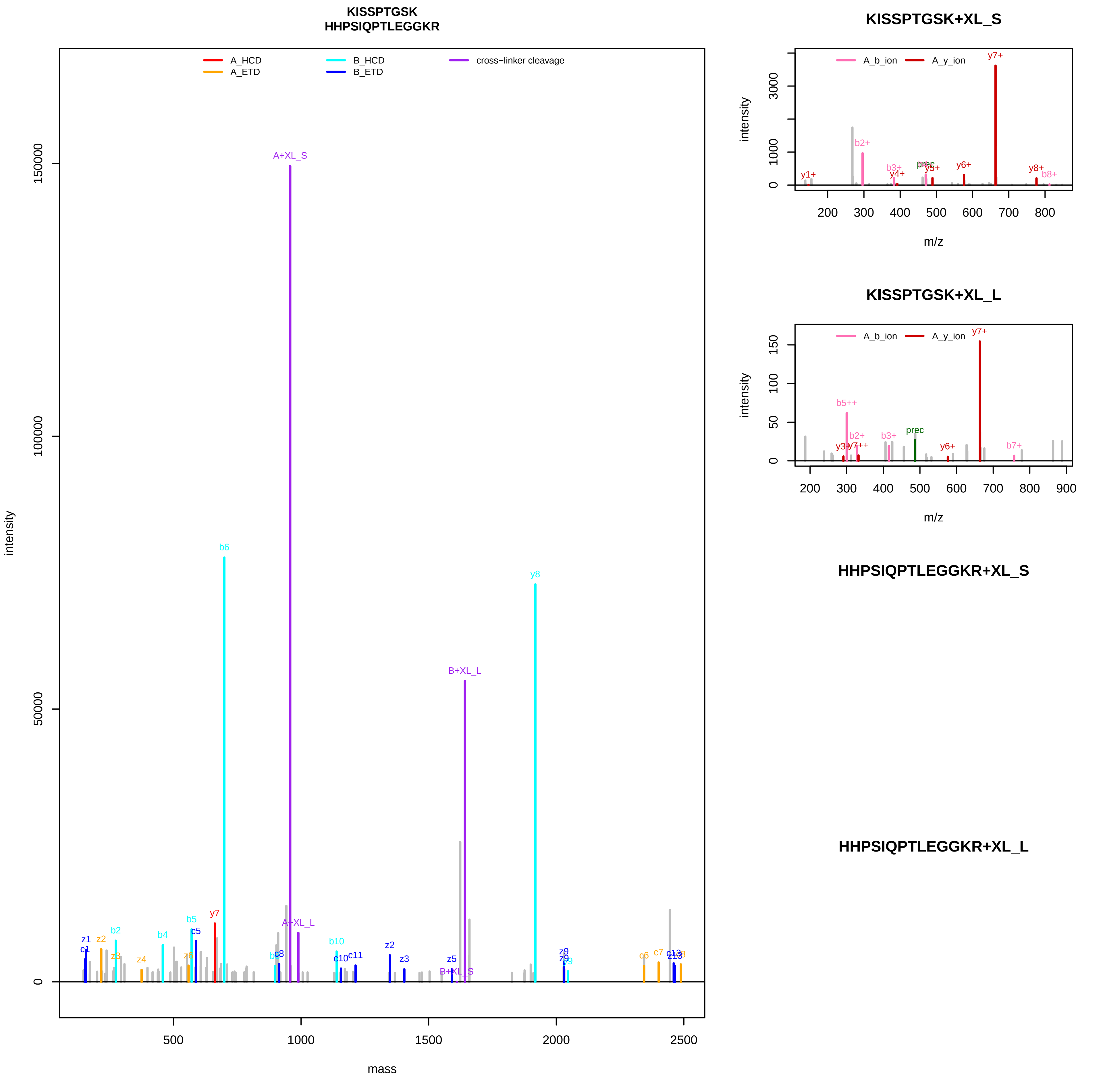
KISSPTGSK
HHPSIQPTLEGGKR
A_HCD
A_ETD
B_HCD
B_ETD
cross−linker cleavage
intensity
mass
0
50000
100000
150000
500
1000
1500
2000
2500
A+XL_S
b6
y8
B+XL_L
A+XL_L
y7
b5
c5
b2
z1
c1
z2
b4
z4
b10
c10
c11
z2
z3
z5
B+XL_S
z9
c6
c7
c13
z13
KISSPTGSK+XL_S
A_b_ion
A_y_ion
y7+
b2+
b3+
y4+
prec
y5+
y6+
y8+
b8+
y1+
intensity
m/z
0
1000
3000
200
300
400
500
600
700
800
KISSPTGSK+XL_L
A_b_ion
A_y_ion
y7+
b5++
b2+
y3+
y7++
b3+
prec
y6+
b7+
intensity
m/z
0
50
100
150
200
300
400
500
600
700
800
900
HHPSIQPTLEGGKR+XL_S
HHPSIQPTLEGGKR+XL_L
